# Are Differences in Physical Activity across Socioeconomic Groups Associated with Choice of Physical Activity Variables to Report?

**DOI:** 10.3390/ijerph15050922

**Published:** 2018-05-05

**Authors:** Ragna Stalsberg, Arve Vorland Pedersen

**Affiliations:** 1 Department of Circulation and Medical Imaging, Faculty of Medicine and Health Sciences, Norwegian University of Science and Technology, Postboks 8905, Trondheim N-7491, Norway; 2 Department of Neuromedicine and Movement Science, Faculty of Medicine and Health Sciences, Norwegian University of Science and Technology, Postboks 8905, Trondheim N-7491, Norway; arve.v.pedersen@ntnu.no

**Keywords:** lifestyle, social position, socioeconomic status, physical activity, activity domains, review

## Abstract

Despite being challenged in recent years, the hypothesis that individuals of higher socioeconomic status (SES) are more physically active than their lower SES counterparts is generally considered a fact. Recent reviews, however, have suggested that differences across groups might be related to which physical activity (PA) domains have been investigated. In the present review, searches for relevant studies were performed in the MEDLINE, ISI Web of Knowledge and SPORTDiscus databases. Search terms included “socioeconomic”, “socio-economic”, “socio economic” and “social class” to meet all variations of the variable “socioeconomic status” in combination with the term “physical activity”. Studies were included when applying the dimensions of intensity, frequency, type/mode, and duration in measuring PA. Fifty-six studies were included and were subsequently split into four PA domains: transport PA (TPA), occupational PA (OPA), housing PA (HPA) and leisure time PA (LTPA). It turned out that the positive relationship held only for LTPA, whereas the relationship was non-existent or even opposite for all other domains. It is concluded that the assumed positive relationship between SES and PA is mainly a relationship between LTPA and SES. It is further suggested that the PA domain should always be considered when studying said relationships.

## 1. Introduction

It has long been assumed that there is an association between socioeconomic status (SES) and physical activity (PA) in that people of high SES are more physically active than those of lower SES (see, for example, [1,2]). Such a difference across socioeconomic groups has been touted as a cause of health-related differences and used to justify advocacy for the introduction of interventions targeted at increasing levels of PA in lower socioeconomic groups [1,2].

More recently, however, several papers have emerged questioning this relationship, among them the reviews by Gidlow, Johnston, Crone, Ellis, and James [3]; Beenackers, Kamphuis, Giskes, Brug, Kunst, Burdorf and Lenthe [4] and Stalsberg and Pedersen [5]. Beenackers et al. [4], in fact, found that in studies reporting occupational PA (OPA), low-SES groups came out as more active, whereas the results were similar across SES groups for active transport. The only domain in clear favor of high-SES groups was leisure-time PA (LTPA). For total PA, the picture was mixed, with about the same number of studies reporting each way. Gidlow et al. [3], although reporting a clear effect of SES when comparing the most extreme (highest and lowest) SES groups, reported relatively mixed results for the remainder of the data. Gidlow et al. [3] discussed problems with the operationalization of the SES variable but reported that education was most commonly used and seemed to produce the most stable relationships. Stalsberg and Pedersen [5] identified similar methodological problems with both variables (PA and SES) as mentioned above and revealed also that more than 40% of studies on adolescents had found no differences in PA across SES groups. A few even reported opposite results with the low-SES group as more active (see [5] for details).

The common denominator of the mentioned studies was that they pointed to variations in relationships across PA domains and argued that differences across socioeconomic groups might be restricted to differences in organized LTPA, whereas other PA domains such as transport PA (TPA), occupational PA (OPA) and housing PA (HPA) had been somewhat overlooked. That there would be a difference in LTPA across socioeconomic groups is perhaps less surprising, considering that individuals of low SES more often have physically demanding occupations, with heavy and repetitive work [6], longer work hours, and evening and nightshift work more often [7]. Thus, individuals of low SES have less leisure time and less energy to participate in LTPA. Furthermore, organized LTPA is often costly, hence further decreasing the possibilities for participation in organized LTPA for low-SES individuals. True enough; studies confirm that individuals of higher SES participate more frequently in organized LTPA. Hence, interventions including organized LTPA may be less helpful to level out social inequalities in health-related variables unless they focus on increasing access for those who cannot otherwise afford it [8].

Taken together, the mentioned findings suggest that reported differences in PA levels across socioeconomic groups might be biased by an undue focus on LTPA. Stalsberg and Pedersen [5] concluded that although a majority of studies reported a positive relationship between high SES and PA, the relationship was far less clear than what was usually touted. Furthermore, high PA among high-SES groups reported in studies was overwhelmingly LTPA, a fact Palma and Assis highlighted [9] in a commentary. These authors argued further that the whole field of research on PA and health was biased by the fact that researchers were all from developed countries and studied variables that were relevant for individuals in such countries. Palma and Assis concluded that the results of such research painted an unrepresentative picture of the field and, thus such findings would be less relevant for developing countries. Del Duca, Nahas, Garcia, Silva, Hallal, and Peres provided an elegant example, of the importance of considering multiple PA domains. In their study, when adding active commuting to the mix of PA, nearly twice as many individuals adhered to PA recommendations than when only LTPA was counted [10].

In addition, comparing only the number of hours, or minutes, of PA across SES-groups does not provide sufficient evidence for conclusions about health issues. Beckvid-Henriksson, Franzén, Elinder, and Nyberg [11] found, for example, that children from low-SES families were more physically active compared with their high-SES counterparts. Despite this fact, they were more often obese and overweight. The authors thus suggested that one should examine other variables such as diet to identify explanations of health differences across socioeconomic groups.

As both Gidlow et al. and Stalsberg and Pedersen have stressed, considerable methodological challenges plague studies of SES and PA [3,5]. Because both SES and PA are notoriously difficult variables to operationalize; their relationship is similarly difficult to establish, and demands considerable attention to numerous mediators (see, for example, [8,10]). Although Gidlow et al. first and foremost discussed challenges related to SES measures [3], Stalsberg and Pedersen, inspired by Rice and Howell [12], underscored the significance of measuring several dimensions of PA [5]—namely *frequency* (the number of PA events during a specific period), *intensity* (physiological effort associated with participating in a particular type of PA), *duration* (time of participation in a single bout of PA) and *type* of activity. In their paper on methodologies used to assess PA, Warren et al. reiterated the argument: that it is difficult to obtain a valid measure of PA [13].

Inspired by the mentioned studies, especially the work of Beenackers et al. [4] who clearly demonstrated the significance of differentiating between domains of PA, the present review set out to investigate whether the assumed positive relationship between SES and PA may have been somewhat overestimated because the majority of studies on the topic have reported data on LTPA. There are two notable differences between Beenackers et al.’s study and the present one. First, Beenackers et al. restricted their study to European adults, whereas we imposed no such restrictions given Stalsberg and Pedersen’s [5] observation of regional differences outside Europe, and given Palma and Assis’ [9] suggestion that developing countries were misrepresented in studies of PA. Second, we attempted to present a more standardized operationalization of PA than did Beenackers et al., by applying Rice and Howells’ criteria [12]; thereby securing data that would be more comparable across studies.

Our review is not a traditional systematic review as far as it does not seek to synthesize or summarize previously reported results. Instead, the aim was to identify variations in findings across individual studies, and to examine whether these might have stemmed from the selection of PA domains investigated.

## 2. Materials and Methods

Computerized searches were conducted in the MEDLINE, ISI Web of Knowledge (ISI) and SPORTDiscus databases to identify all relevant articles published from 2000 to 2010. A subsequent search was performed, that encompassed more recent papers (published between 2010 and 2014). To include all variations of the variable “socioeconomic status”, the search terms “socioeconomic”, “socio-economic”, “socio economic” and “social class” were used in combination with the term “physical activity”. To exclude studies on children and adolescents, the search limit “19 years plus” was imposed upon the search performed in MEDLINE, and the terms “grownups” and “adult” were added in the ISI search. The search in SPORTDiscus was performed without pre-set boundaries.

The first, relatively open, search (Search 1) returned 1225 articles, many of which, we quickly realized were not relevant whatsoever. We therefore added further limitations, as shown in the search criteria of MEDLINE/PubMed (Search 2) presented in Table 1, which after proving their worth, were applied to all subsequent searches. We have presented Search 1 in Table 1 to illustrate the differences between the two search strategies. By imposing the additional limitations, we avoided sifting through roughly 500 irrelevant titles and abstracts, as well as possible several hundred others in subsequent searches. Ultimately, slightly more than 3400 titles and abstracts were examined to identify studies that would meet the inclusion criteria, and, of those, 385 potentially relevant articles were thoroughly investigated to establish their eligibility according to the criteria.

To be included in the review articles had to report empirical studies with original data, including data from national surveys, that represented adult participants of both genders; address the relationship of SES and PA in their titles or abstracts; apply Rice and Howells’ dimensions in measuring PA (i.e., intensity, frequency, type or mode and duration); and be written in English. Studies with the aim of investigating physical inactivity (PIA) that applied an adequate method of assessing the level of PA, were included. 

By contrast, articles were excluded if they reported studies with samples of disabled individuals or people with diseases exclusively; reported studies on motor skills; were doctoral **theses,** descriptive or theoretical papers, abstract of books or proceedings, conference papers or reviews; reported intervention studies with only either low- or high-SES groups; reported studies with single-gender samples; reported studies using the SES of the respondents’ parents (in the case of for example university students); reported studies that applied fewer than four of the mentioned dimensions in measuring PA (i.e., intensity, frequency, type or mode and duration); or primarily addressed methodological questions.

Each of the databases searched offered schemes for imposing limits on the searches. To ensure that the selections of articles were based on the same criteria, some limitations had to be imposed during the reading process and others using pre-set limitations offered by the database. Limitations imposed on the searches appear in Table 1. The first author performed all searches, and both the two authors discussed the few articles whose eligibility was uncertain and determined their merit according to the criteria.

### 2.1. Data Extraction

From the studies included in the sample, data relevant to the present review were extracted, and registered the variables *aim of study*, *design*, *sample characteristics* (including gender, age, and nationality), *measures of SES*, and *outcome/conclusions*.

Next, the various measures of SES were categorized by education, income, occupation, neighborhood or other if none of the mentioned categories pertained. In addition, less precisely defined variables (e.g., when income was dichotomized as low or high) were registered. Measures of PA were registered according to the four valid measurement dimensions (i.e., duration, frequency, intensity, type, or mode of PA). Phrases similar to “for at least 30 min at a time” were coded as duration. The question of whether the exercise could be regarded as vigorous or moderate was recorded as a measure of intensity. In some studies, authors had pre-calculated intensity by type of PA, particularly when the terms “moderate” and “vigorous” activity were used or when Ainsworth’s code schemas, which classify specific PA by rate of energy expenditure as the Metabolic Equivalent for Tasks (METs) [14], was cited.

### 2.2. Analysis

Papers were thoroughly reviewed for the directions of relationships reported, although-based also with attention to primary tendencies in the results. The categories of relationships, denominated as positive (i.e., high-SES groups being more active), negative (i.e., low-SES groups being more active), mixed (both high- and low SES being more active according to type of activity or SES measure) and no relation, were then sorted by continental affiliation (i.e., Europe, North America, South America, Asia, Africa, and Oceania). To minimize the complexity of presentation, studies of PIA reporting more inactivity in lower-SES groups were registered as having reported positive relationships. Studies reporting more inactivity among higher-SES groups were thus categorized as having reported a negative relationship.

Within each geographical cluster, the frequency of studies with positive, negative, mixed or no relationship were recorded for each SES measure applied. Education was applied as a measure of SES in 16 European studies, 10 of which demonstrated a positive, one a negative, four a mixed, and one a non-existent relationship. A similar procedure was performed for the different domains of PA that emerged during the analysis. If results referred to PA guidelines or to several domains of PA combined, they were recorded in separate groups.

Although results from studies of PIA were included in the analysis (more inactive groups considered less active) they were not analyzed as a freestanding group. Studies investigating gender differences were identified and analyzed both in terms of the primary (i.e., total) sample and as males and females separately.

## 3. Results

The searches returned 56 relevant studies, which were subsequently included in the final sample. Table 1 presents the search strategies and results. Above all, the outcome revealed complexity in the association between SES and PA among adults that adds important nuances to common assumptions about the relationship of SES and PA.

The sample included studies representing 30 nations in total; 22 studies were European, 11 were Asian, nine were North American, eight were South American, five were Oceanian (i.e., Australian) and one was African (i.e., Nigerian). Almost three out of four (41) of the articles had been published during the second half of the period (2008–2014) and a third during the past 2 years. The samples varied widely, from 276 [15] to 55,151 [16], and women were slightly overrepresented in nearly every study. Regarding age composition, the studies’ samples were relatively similar; at the extremes, one had a mean age of 22.4 years [17,18] and the other a mean age of 75 years [19]. Except for samples from a few studies with slightly narrower age ranges, samples ranged from 18 to 65 or from 16 to 75 years. Three North American studies had particularly high-age samples of 53–97, 65–80+ and 50–79 years. Most of the studies were based on data from either interviewer- (e.g., telephone) or self-administered questionnaires, with the notable exceptions of van Dyck et al. [20], who complemented their data using accelerometers, and Golubic et al. [21], who combined self-reporting with heart rate and movement censoring. The vast majority of studies (*n* = 48) used education as an SES measure, whereas occupation was the most rarely applied measure (*n* = 14). Usually, two or more but no more than five measures were applied in each study to establish SES.

Three fourths of all studies analyzed reported results related to all four dimensions of PA. In 30 of those studies, PA was operationalized as a rate of energy expenditure (e.g., total energy expenditure (TEE) or METs). When results from fewer than four dimensions were reported, intensity was the dimension most often excluded from analysis.

In what follows, four tables are presented describing studies in the sample. Each table describes a different direction of relationships; Table 2 includes studies demonstrating predominantly positive relationships (i.e., high SES groups reported to be more active than low-SES groups), Table 3 includes studies with negative relationships (i.e., low-SES groups reported to be more active than high-SES groups), Table 4 includes studies reporting no relationship, and Table 5 includes studies demonstrating mixed relationships (i.e., positive, negative and non-existent) within the same study. Each table lists articles according to continental affiliation and, thereafter, by year of publication.

### 3.1. Directions of Relationships: Geographical Region, Period of Publication, SES Measure and Age

Of all 56 studies in the sample, fewer than half (23) reported a predominantly positive relationship between PA and SES. Nine studies reported a primarily negative relationship (low SES more active), whereas three studies showed no relationship at all. The remaining 21 studies reported mixed results.

Only one of the 11 Asian studies [42] reported a positive relationship between PA and SES (i.e., greater likelihood of PIA in lower-SES groups), whereas approximately half of the studies from all other continents demonstrated positive relationships.

Over time, although the proportion of studies showing positive results remained constant, the group of studies showing mixed results diminished at the expense of studies showing negative or no relationships.

The results of our analysis provide no evidence that the choice of SES variable affects the direction of the relationship between PA and SES in adults. No marked differences emerged in the use of SES measure by continental affiliation, either.

Using the mid-range of the individual age range in each sample, except when mean age was the age-related information given, we calculated the arithmetic mean, mode and median of age in each group of studies categorized according to the direction of relationship between PA and SES (Three studies were excluded from these calculations due to limited information on age (i.e., lowest age only)). The group of studies demonstrating positive relationships had a slightly higher mean, mode, and median age (i.e., 48.5, 45 and 45 years, respectively) than the other groups. Conversely, the group of studies demonstrating mixed relationships between PA and SES had the lowest mean, mode, and median age (i.e., 41.6, 40 and 40 years, respectively).

### 3.2. Physical Activity Domains

**Figure 1:**
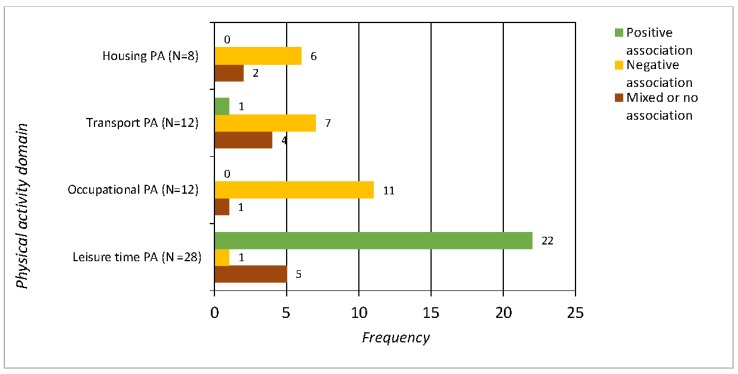
Results demonstrating positive, negative, mixed or no association between SES and PA within PA domains. Occasions, in which a domain has been studied (some studies include more than one domain). Seventeen studies reporting either PIA or total PA level without separating different PA domains are not included in this figure.

All studies included in the present review presented data on the type or mode of PA, sometimes referred as “PA domains” (i.e., LTPA, OPA, TPA and HPA), according to which they were categorized. For most studies in which the term “domain” was not used, it was still possible to assign the type of PA to a domain. Sports, exercise and walking for recreation were classified as LTPA, for example, whereas gardening was classified as HPA. By enumerating the frequency at which the different domains were studied, a preponderance of LTPA was observed either alone or in combination with other domains. Studies had examined OPA and TPA equally often, albeit far less than LTPA (see Figure 1 for details).

Categorizing the studies revealed a clear tendency of a positive relationship between PA and SES in the LTPA domain but not necessarily in other domains. In 22 of the 32 studies addressing LTPA [16,19,24,25,28,29,30,33,38,40,41,43,55,57,59,60,61,64,66,67,68,69], a positive relationship with SES was found, whereas a negative relationship was found in only one study [44]. The remaining nine studies [21,32,45,46,51,52,53,54,58] reported less clear answers due to differences dependent upon gender, SES-measure, or other confounding effects. Nine of the 11 studies that included the OPA domain demonstrated negative relationships between PA and SES [15,45,55,57,60,61,65,66,67] whereas none of the studies including the OPA domain demonstrated a positive relationship between OPA and SES. In two studies [55,70], results were mixed due to differences across gender. Studies that included the TPA domain seemed to similarly demonstrate negative results; nine such studies [16,20,46,47,57,61,64,67,68] demonstrated negative relations, whereas three demonstrated non-existent or negative relationships [17,18,69], if not both. Of the eight studies examining HPA and SES, none demonstrated a positive relationship, although four demonstrated negative relationships [57,60,67,69], and four others demonstrated non-existent or mixed relationships related to gender differences [25,54,59,61], as illustrated in Figure 1.

### 3.3. Effects of Gender

An analysis of a subgroup of 26 studies reporting gender-specific results revealed that the relationship between SES and PA was positive for both men and women in the LTPA domain. The mentioned relationship between SES and OPA remained negative for men (low SES more active) but might have been somewhat less established in women. For the remainder of the domains (i.e., TPA and HPA), no clear trend was evident across the studies.

## 4. Discussion

In the present review, only 23 of the 56 studies (41%) found that individuals of higher SES were more physically active than their low-SES counterparts, whereas nine studies reported the opposite (individuals of lower SES were more physically active). For 24 studies (43%), resolution is still wanting, in that they report either no effect of SES on PA or mixed effects with some variables favoring high SES and others falling on the side of the lower-SES population.

When the results were organized by PA domains, a very clear picture emerged. Out the 32 studies that reported LTPA, 22 concluded that individuals with higher SES were more active, whereas that relationship appeared only once in the 33 studies when other PA domains examined (Figure 1). Regarding the other domains (i.e., HPA, TPA and OPA), an inverse relationship appeared for as many as two-thirds of studies, indicating that individuals from lower-SES groups were more physically active. In nine studies, no relationship was found between PA and SES.

Furthermore, when results were organized according to the respective domains, a few important nuances surfaced. LTPA was positively related to SES irrespective of gender, whereas the OPA-SES relationship was positive for males and negative for females. The other relationships (i.e., TPA-SES and HPA-SES) remained unclear, however.

Our results show, as with Stalsberg and Pedersen [5], Gidlow et al. [3] and Beenackers et al. [4] before, that the relationship between PA and SES is not as clear-cut as assumed. More importantly, the results support Beenackers et al.’s [4] findings that the relationship between PA and SES depends upon which PA-domains are measured. Thus, our findings upheld our hypothesis. At the same time, although we had limited data from developing countries, our results seem to support Palma and Assis’ [9] argument that studies’ undue focus on LTPA would misrepresent PA levels among populations in such countries. To that argument, we can add that the same would apply to the low-SES population of developed countries. Furthermore, the focus on PA in interventions, although certainly warranted, has obscured other variables not under the control of individuals. For example, the PA level of individuals of low SES likely suffers from their living in areas with less access to parks [71], or with less neighborhood walkability [72] and their health is also negatively affected by the cost of healthy food compared to that of junk food [73].

What the present results may indicate is that although individuals of lower SES have fewer financial resources to engage in leisure activities, they are more physically active than has been assumed when other PA domains (e.g., OPA and TPA) are taken into consideration. In, for example, Del Duca et al. [10] mentioned earlier, many individuals who were otherwise categorized as inactive, in fact, met recommendations for PA when data on TPA were included as opposed to when only LTPA was counted. It is reasonable to assume that people of lower SES have less surplus energy to be physically active during their leisure time, because of the physical strain of their work [74]. Moreover, there is reason to believe that people of higher SES are more active in their leisure time, out of necessity, because they are less physically active at work [67], and not merely because of their ability to finance their activities.

The various operationalizations of the SES variable in previous studies have complicated comparisons across studies (see Gidlow et al. [3], and Stalsberg and Pedersen [5] for some more detail). In the present review, as education was the predominant variable for establishing SES among included studies, and the PA variable was held more stringent by the inclusion criteria, thus it secured a more homogenous batch of studies, the picture becomes clearer. The previously touted relationship between SES and PA is mainly a relationship between higher education and LTPA.

Our results also suggest that studies on PA, including those investigating relationships with SES, have largely focused on LTPA, often in the form of registered sports participation, membership in sport clubs, and the like. That trend was apparent in all but five studies in our sample, and in 15 studies, LTPA was the sole variable. Studies of OPA, TPA and HPA remain scarce and have often been hampered with methodological inadequacies that blur the results. The reason for such bias could be that the four dimensions of PA (mentioned earlier) are easier to report in sports and other forms of LTPA and, even that the PA-questionnaires predominantly used are better adjusted for reporting such activities.

The mentioned methodological consequences of over-generalizing results of LTPA-oriented studies could partly explain many of the observed differences in our dataset. For example, among Asian studies, only one of 11 studies [42] demonstrated positive relationships between PA and SES (higher SES were less inactive) compared with approximately half of the studies from all other continents combined. Such a finding suggests either that the relationship between the SES and PA differs for Asians compared with the rest of the world or, more likely, that the European and American studies have placed undue focus on LTPA compared with other domains.

In studies that reported less clear or even negative relationships, observed gender-based differences also arguably coincide with the reality that women less often than men engage in sports [75], more often than men engage in household activities [76] and have less physically demanding occupations [77,78] than men do. Thus, no relationship emerged between PA and SES for females in our results.

The trend, albeit unclear, that studies including older participants more often demonstrate positive relationships between SES and PA could relate to the fact that older individuals have more leisure time than younger ones. Furthermore, when studies have included groups of retirees, they have run the risk of underreporting OPA as well as TPA to and from work that would otherwise shift total PA in the direction of the low-SES group.

The change in the relationship over time, also unclear, that more recent studies more often have demonstrated negative relationships could partly derive from the fact that those studies, compared with previous ones, included other PA domains instead of focusing solely on LTPA. Moreover, the trend of studies being more geographically diverse in recent years might have served to shift the focus away from LTPA.

When measuring LTPA, and drawing conclusions about PA as a result, low-SES groups have appeared to be less physically active than they are, whereas the PA levels of high-SES groups have been overestimated. In addition, as Palma and Assis [9] have underscored, developing countries are misrepresented as having less physically active populations than developed countries because the former have far more physically demanding, time-consuming work that leaves less time for leisure activities, both due to less leisure time and greater fatigue after work, hence their reduced inclination to engage in PA.

Another factor could be that studies of PA and SES are typically designed and conducted by individuals who belong to high-SES groups (e.g., researchers and physicians), which are characterized by their higher education, higher income, and less physically demanding occupations [79]. A social group holding the power to define and value or rate a social phenomenon might have the misfortune to disregard their own preconceptions and introduce bias as a result.

Recent studies have argued that sedentary time might be a better indicator of health risk than lack of PA is [80,81]. Furthermore, it has been suggested that not even increased levels of physical exercise, sometimes mistaken for increased levels of total PA, can compensate for the declining levels of everyday PA, tentatively termed “daily life physical activity” (DLPA) by Stalsberg and Pedersen [11]) (cf. [82,83]). The over-eager focus on LTPA in scientific studies might have masked the lack of DLPA and thus prompted the underestimation of public health risks.

When studying PA to be able to offer health advice, researchers should remember that the level of PA is but one of several variables that determine an individual’s health. As a case in point, Beckvid Henriksson et al. [11], found that the most physically active group – in their case, the low SES group—was also the one with the poorest health. In response, those authors suggested differences in diet across groups as another variable relevant to explaining their findings (see also [84]).

The present study clearly involved limitations. Above all, our results cannot falsify the claim that individuals of higher SES are more active, which was not our aim in the first place. Furthermore, our results do not support conclusions about the total PA levels of any SES-group, since most studies do not report all PA-domains. Even if individuals of low SES are more physically active than they have been credited to be, we do not know whether that trend would contribute positively to their health, for all PA is not necessarily equally healthy. In fact, much of their work PA, might even be harmful. Last, we did not include data on sedentary time for any of the groups, which makes it impossible to draw conclusions about any health-related issues, as they depend upon both total PA and accumulated sedentary time.

What our results contribute, however, is that the findings of studies seem to have varied across PA-domains and that the entire field of research on PA seems to have given undue attention to LTPA. Thus, results might become less clear when other domains are added to the mix. That possibility indicates directions for future studies seeking to respond to the questions that remain unanswered. More practically, researchers should report all PA-domains and account for sedentary time so that the variables can be balanced against each other.

## 5. Conclusions

The assumed relationship between PA and SES is mostly a relationship between LTPA and high SES. No such relationship or a negative relationship between PA and SES for all other PA domains exists, which indicates that individuals from low-SES groups are more active. Whether the high- or low-SES group is more physically active in total remains unclear and is difficult to determine with any certainty based on available data. In any case, no comparison of PA across SES groups should be made without accounting for not only total LTPA, as is currently common, but also total PA. Developing countries and the low-SES group might also have been misrepresented in studies on PA. Those populations might be more physically active than they have been credited to be, the misconception of which is due perhaps to the fact that researchers most often come from high-SES populations in developed countries. That finding has consequences for practitioners targeting low-SES populations with interventions that attempt to increase their PA levels, and we suggest that researchers and practitioners should look beyond the mere amount of PA for other variables that can explain health-related differences across SES groups.

## Figures and Tables

**Table 1:** Search strategies and findings.

Search Strategy		Articles Identified	Potentially Relevant Articles ^1^	Articles Included
MEDLINE/PubMed 2000–2010, humans, English, 19 years plus				
1	Physical activity and (socioeconomics or socio-economic or socioeconomic or socio economic or social class))	1211	not assessed	-
2	*Physical activity and (socioeconomics or socio-economic or socioeconomic or socio economic or social class)) not (disease or depress or injury or pregnant or neonatal or adiposity or cardiovascular or cancer or kidney or iron or schizophrenia or vitamin or calcium or herbal or osteoporotic or rheumatoid or personality or microbial or lipoprotein or lipid or sleep or menstrual or glucose or insulin or coronary or schistosomiasis or diabetes)*	725	136	18
MEDLINE/PubMed 2010–2014, humans, English, 19 years plus				
	*Equal to search 2 in Medline*	800	64	12
SPORTDiscus 2000–2010				
1	(Physical activity) and (socioeconomics or socio-economic or socioeconomic or socio economic or social class))	262	69	6
SPORT DISCUS 2010–2014				
	*Equal to search 1 in SPORTDiscus*	360	25	5
ISI Timespan = 2000–2010. Databases = SCI-EXPANDED, SSCI, A&HCI.				
1	(Physical activity and (socioeconomics or socio-economic or socioeconomic or socio economic or social class) and (adult or grown up))	260	43	2
ISI Timespan = 2010–2014. Databases = SCI-EXPANDED, SSCI, A&HCI.				
	*Equal to search 1 in ISI*	1007	48	13
TOTAL				56

1 The number of potentially relevant articles refers to the number prior to a control of duplicates. Duplicates identified in MEDLINE were deleted from the list if identified in ISI or SPORTDiscus. Articles identified in ISI were deleted if identified in SPORTDiscus.

**Table 2:** Studies investigating PA in adults across SES. Positive relationships.

Study	Aim and *Study Design*	Sample	Measures of SES ^1^	Outcome/Conclusion
Bernstein et al., 2001	Describe the distribution of PA. *Questionnaires/measures of weight and height*	*n* = 3410 M: 1707 F: 1703 Age: 35–74 SWITZER-LAND	Education: + Income: − Occupation: − Neighborhood: − Other: −	Sedentarism (related to total energy expended) is more prevalent in (…) W and lower SES persons [22]
Bertrais et al., 2004	Evaluate the characteristics of subjects meeting public health PA recommendations. *Questionnaire*	*n* = 7404 M: 3404 F: 4000 Age: 45–68 FRANCE	Education: + Income: − Occupation: − Neighborhood: + Other: −	In W, but not M, education level was positively related to meeting Public health recommendations (PHR) (related to METs). Resident location was not related to the probability of meeting the PHR in M, whereas W who did not live in an urban pole were more likely to meet the PHR compared with women who did [23]
Kamphuis et al., 2008	Examine the contribution of neighborhood, household, and individual factors to SES inequalities in sports participation in a multilevel design. *Postal survey*	*n* = 3839 M: 1836 F: 2003 Age: 25–75 HOLLAND	Education: + Income: + Occupation: − Neighborhood: + Other: deprivation	The lowest educated and lowest income group were most likely to report no sports participation. Significant clustering of no sports participation within neighborhoods. Two out of three indicators of material deprivation (crowding or having financial problems) and all three indicators of social deprivation increased the likelihood of doing no sports. In addition, these factors showed higher prevalence among lower SES groups [24]
Borodulin et al., 2008	Investigate the associations of age and education with types of LTPA. *Self-reported questionnaire*	*n* = 4437 M: 1940 F: 2497 Age: 25–64 FINLAND	Education: + Income: − Occupation: − Neighborhood: − Other: −	Education was directly associated with conditioning and overall LTPA in M and W, but no association was found with daily PA. For both M and W, low education group reported significantly less conditioning activity and overall LTPA than the middle and high education groups [25]
Kwaśniewska et al., 2010	Analyze the epidemiology of TPA and investigate the relationship between TPA and SES and lifestyle. *Questionnaire*	*n* = 7280 M: 3747 F: 3533 Age: 20–74 POLAND	Education: + Income: + Occupation: − Neighborhood: − Other: −	Prevalence of walking/cycling less than 15 min/day was the highest among those with secondary education (both M and W), with the lowest income in M and with the monthly income 130–260 Euros/month in W. Active transportation lasting 15+ min/day was most prevalent in M and W with monthly income above 260 Euros/month. Among both M and W commuting 30+ min/day there was a domination of persons with university education [26]
Stringhini et al., 2011	Examine whether health behaviors are equally important mediators of the SES-health associations in different cultural settings. *Questionnaire*	*n* = 30,933 M: 21,906 F: 9027 Age: 35–55 UK/FRANCE	Education: + Income: + Occupation: + Neighborhood: − Other: −	The difference in prevalence between highest and lowest occupational group was 15% for being PIA. Participation in the lowest occupational group compared to those in the highest were more likely to be (…) PIA [27] (Only Whitehall II, phase I (the British study) is included due to the PA measure criteria)
Łobaszewski et al., 2011	Evaluate the prevalence, socio-demographical patterns and behavioral characteristics of LTPA. *Questionnaire*	*n* = 15,000 M: unknown F: unknown Age: 45–64 POLAND	Education: + Income: + Occupation: − Neighborhood: − Other: −	% of persons engaging in walking in their leisure time was highest in higher income groups. In the lower income SES groups, this proportion was significantly lower. 28.7 of respondents with higher education participated in moderate exercises, 18.2% with secondary education and 11.2% of those with primary or vocational education. 27.8% with the highest income performed moderate PA, but significantly lower for those with a lower income. Strong correlation between education and vigorous PA; those with higher education participated significantly more than those with lower education did. A similar correlation was observed for the income variable. Those of lower or medium SES engaged in vigorous exercises significantly less often than those with higher income [28]
Borodulin et al., 2012	Explore associations of education and income with BMI and study the mediating pathways through health behavior. *Questionnaire*	*n* = 3258 M: 1555 F: 1703 Age: 25–75 FINLAND	Education: + Income: + Occupation: − Neighborhood: − Other: −	Significantly positive relationships found between education and LTPA and between income and LTPA for M and W [29]
Ord et al., 2013	Examine the extent to which green space is a venue for PA and if this could account for SES health inequalities in green neighborhood. *Survey*	*n* = 3679 M: 1621 F: 2058 Age: 16–75+ SCOTLAND	Education: − Income: + Occupation: − Neighborhood: − Other: −	An independent, positive association between household income and meeting the recommended walking guidelines and participation in green PA [30]
Uijtdwilligen et al., 2014	Examine the longitudinal of person-related factors with PA behavior in young adults. *Semi-structured interview*	*n* = 499 M: 248 F: 251 Age: 21–36 HOLLAND	Education: − Income: − Occupation: − Neighborhood: − Other: employment	M and W having no paid work spent significantly more time in Moderate PA than those working full time. Full-time working M spent significantly more time in vigorous PA than those without paid work. W: No association [31]
Marques et al., 2014	Identify correlated factors that explain the recommended level of LTPA among Portuguese adults. *Questionnaire*	*n*= 2166 M: 972 F: 1194 Age: 31–60 PORTUGAL	Education: + Income: − Occupation: + Neighborhood: − Other: −	For M, those with middle SES (OR = 1.47, 95% CI: 1.04–2.06, *p* = 0.028), high SES (OR = 1.88, 95% CI: 1.35–2.62, *p* < 0.001), had a higher and significant tendency for meeting PA recommendation in leisure time. For W, middle SES (OR = 1.40, 95% CI: 1.04–1.89, *p* = 0.026), middle level of education (OR = 1.41, 95% CI: 1.05–1.89, *p* = 0.023) were significantly associated with meeting PA recommendations during leisure time. For W, educational level was not significant when incorporated into the multivariate analysis [32]
Satariano et al., 2002	Examine the extent to which differences in LTPA are associated with differences in living arrangements. *Questionnaire*	*n* = 2073 M: 842 F: 1231 Age: 53–97 USA	Education: + Income: + Occupation: − Neighborhood: + Other: employed	Level of education was an important factor for both W and M. Those who engaged in higher levels of LTPA in both the full sample and among the married W were more likely to have had more than 12 years of education. Odds of participation were also elevated among W with more than 12 years of education. Engagement in highly vigorous PA compared to brisk PA also was elevated among W with more than 12 years of education (associations of LTPA and income/neighborhood are unknown) [19]
Huston et al., 2003	Examine associations between perceived neighborhood characteristics, access to places for PA, and LTPA. *Phone survey*	*n* = 1796 M: 680 F: 1116 Age: 18–65+ USA	Education: + Income: + Occupation: − Neighborhood: + Other: −	The % reporting any PA increased with increasing education level and with increasing income. The % engaging in recommended PA was higher in higher education groups and increased with increasing income. Although neighborhood characteristics were positively associated with engaging in any LTPA, these associations did not remain significant after adjusting for socio-demographic and other environmental factors. Neighborhood trails were also positively associated with engaging in PA, even after adjusting for socio-demographic and other environmental factors [33]
Ashe et al., 2008	Determine the proportion of elders who achieved a recommended amount of PA, and identify variables associated with meeting guidelines. *Telephone interview*	*n* = 24.233 M: 14,539 F: 9694 Age: 65–80 CANADA	Education: + Income: + Occupation: − Neighborhood: − Other: −	Higher proportions of people in the *No chronic disease* group met the PA guidelines if there was a higher level of education or income. Respondents in the highest income and education categories in the *Chronic disease* group attained the same proportion as the overall mean for the *No chronic disease* [34]
Azagba & Sharaf, 2014	Examine LTPIA and its correlates among older Canadian adults. *Questionnaire*	*n* = 45,265 M: 22,814 F: 22,451 Age: 50–79 CANADA	Education: + Income: + Occupation: − Neighborhood: − Other: −	Significant association with being PIA. Education: postsecondary (OR = 0.62, CI = 0.57–0.68), some postsecondary (OR = 0.68, CI = 0.58–0.80) and secondary (OR = 0.81, CI = 0.73–0.91) are less likely to be PIA relative to those with less than secondary education. Income: only the high and low middle-income categories are significantly different from low income. Those in the high-income category are less likely to be PIA than the low-income category (OR = 0.90, CI = 0.81–1.00) [35]
Dias-da-Costa et al., 2005	Measure the prevalence of PIA during leisure time, and identify variables associated. *Questionnaire*	*n* = 1968 M: 846 F: 1122 Age: 20–69 BRAZIL	Education: + Income: + Occupation: − Neighborhood: − Other: household	Schooling and economic level were inversely related to low LTPA [36]
Azevedo et al., 2007	Explore the association between gender and LTPA, and study a variety of variations associated with PA. *Questionnaire*	*n* = 3100 M: 1344 F: 1756 Age: 20–70 BRAZIL	Education: + Income: − Occupation: − Neighborhood: − Other: economic level	M with high education presented 75% lower risk of scoring zero in comparison to those with low education. Among W, this difference was 35%. Economic level showed a clear dose-response positive association with the PA score among W. Those in the least wealthy group (‘E’) presented 110% increased prevalence of score zero in comparison with those from level “A”. Among M, groups “C”, “D” and “E” presented comparable prevalence of subjects scoring zero, approximately 60% higher than M from the “A” level [37]
Reis et al., 2013	Examine the association between walkability and PA outcomes, and the effect of income on the relation between walkability and PA in adults. *Questionnaire*	*n* = 697 M: 334 F: 363 Age: 18–65 BRAZIL	Education: − Income: + Occupation: − Neighborhood: + Other: numbers of cars, children	No interactions between walkability and income were found. Leisure- time moderate-to-vigorous PA ranged 12.2–19.3% in low income areas, and 25.3–35.8% in high-income areas. Neighborhood income was independently associated with leisure- time moderate-to-vigorous PA (OR = 1.70, 95% CI = 1.06, 2.74, *p* = 0.029) [38]
Brown & Siahpush, 2006	Investigate predictors of being sedentary. *National Health Survey*	*n* = 16,243 M: 7600 F: 8643 Age: 18–60+ AUSTRALIA	Education: + Income: + Occupation: + Neighborhood: Index of relative SES	Low education level, blue-collar occupation, low income, and area social disadvantage were all significant predictors of sedentary behavior. Significant relationships between all SES variables and PA levels in both M and W. All indicators of low SES are powerful individual contributors to being sedentary [39]
Cerin et al., 2008	Identify individual, social, and environmental contributors to individual- and area-level differences in LTPA across SES. *Questionnaire*	*n* = 2194 M: 790 F: 1404 Age: 20–65 AUSTRALIA	Education: + Income: + Occupation: − Neighborhood: + Other: employment status, household	Respondents with a medium household income had 12.9%, and those with a high household income had 23.5% higher mean values of walking for recreation than respondents with a low household income. Compared to the SES reference categories, individuals with a secondary education, with medium household income, and living in a medium-income neighborhood would report 33.5% more recreational walking due to differences in the examined mediating variables. The mediated difference in mean walking between the lowest and highest SES categories was 53.9% [40]
Gearon et al., 2013	Ascertain the contribution of specific dietary elements and LTPA to variations in obesity with education. *Questionnaire*	*n* = 30,630 M: 12,141 F: 18,489 Age: mean 55 AUSTRALIA	Education: + Income: − Occupation: − Neighborhood: − Other: −	Those with lower educational attainment appeared less likely to engage in high levels of LTPA for both M and W [41]
Mabry et al., 2012	Identify sociodemographic, anthropometric, and behavioral correlations of occupational, transport and leisure-time inactivity (OPIA, TPIA and LTPIA), and sitting time among adults in Oman. *Questionnaire*	*n* = 1335 M: 591 F: 744 Age: mean 36.3 OMAN	Education: + Income: − Occupation: − Neighborhood: − Other: work status	M: no significant association with OPIA or TPIA. Significantly higher odds of LTPIA for lower education (*p* = 0.03), and for not employed vs. employed (*p* < 0.05). F: no significant association with OPIA or TPIA. OR of LTPIA were 1.8 higher for not employed [42]
Adeniyi & Chedi, 2010	Explore the SES and demographic predictors of PA in pre-retired and retired in Nigeria. *Questionnaire*	*n* = 532 M: Unknown F: Unknown Age: 28–68 NIGERIA	Education: + Income: + Occupation: − Neighborhood: − Other: job duration	For both the retired and pre-retirement civil servants (…) current monthly income and job duration significantly predicted their engagement in mod PA. The lowest income group and the respondents with shortest job duration had significantly lower engagement than the higher SES groups [43]

^1^ The symbols +/− indicates whether the particular SES measure is used in the study (+) or not (−).

**Table 3:** Studies investigating PA in adults across SES. Negative relationships.

Study	Aim and *Study Design*	Sample	Measures of SES ^1^	Outcome/Conclusion
Van Dyck et al., 2010	Investigate whether neighborhood walkability is positively associated with PA and whether this association is moderated by neighborhood SES. *Questionnaire + accelerometer*	*n* = 1166 M: 558 F: 607 Age: 20–65 BELGIUM	Education: − Income: − Occupation: − Neighborhood: annual household Other: −	Living in a high-SES neighborhood was associated with significantly less walking for transport and more motorized transport. The accelerometer measured less activity (min/day) in the high SES neighborhood [20]
Guessous et al, 2014	Examine the association of cardiovascular risk factors, biomarkers, and SES factors with PA. *Questionnaire*	*n* = 9320 M: 4619 F: 4659 Age: 35–74 SWITZER-LAND	Education: + Income: + Occupation: + Neighborhood: − Other: −	High education level subjects had lower activity than subjects with low education had. Compared to the category of non-manual, managerial or independent labor, all other categories had higher 3+ MET-minutes per week, especially those with manual labor occupations [44]
Wolin et al., 2008	Explore potential variation in the OPA–LTPA relation across gender and socioeconomic position strata. *Survey*	*n* = 5448 M: 2550 F: 2898 Age: 18–70 USA	Education: + Income: − Occupation: − Neighborhood: − Other: −	There was no association between education and LTPA. The association remained non-significant after adjusting for covariates and in gender-stratified multivariable models. Significant inversely association between education and OPA [45]
Hearst et al., 2013	Investigate the relationships between neighborhood-level sociodemographic context, individual level sociodemographic characteristics and walking for leisure and transport. *Questionnaire*	*n* = 550 M: 118 F: 432 Age: 26–70 USA	Education: + Income: − Occupation: − Neighborhood: + Other: free lunch	Those w/least resources did most walking overall. Those from the highest two levels of resources or least disadvantaged neighborhoods had fewer minutes of TPA walk as compared to those coming from the least resourced or most disadvantaged neighborhoods. There were no differences in LTPA walk by neighborhood characteristics. There was no significant difference in walking by education level although there was a trend for less LTPA walking for individuals reporting at least a college education. Finally, those respondents who did not report qualifying for free or reduced lunch had fewer minutes of TPA walking as compared with those that did qualify for free/reduced lunch [46]
Kienteka et al., 2014	Analyze the association between personal and behavioral aspects in TPA bicycling and LTPA bicycling in adults. *Questionnaire*	*n* = 677 M: 317 F: 360 Age: 18–65 BRAZIL	Education: + Income: − Occupation: − Neighborhood: − Other: work status, assets	After adjusting for all confounding variables, those of low SES (PR = 5.00; 95%CI: 1.65–15.17; *p* = 0.006), reported using a bicycle for TPA more frequently [47]
Fogelman et al., 2004	Investigate the accuracy of self-perception of participation in PA, and the correlations of PA with background factors. *Questionnaire*	*n* = 276 M: Unknown F: Unknown Age: 20–65 ISRAEL	Education: + Income: + Occupation: − Neighborhood: − Other: −	Subjects with fewer years of education engaged in more OPA, however, the differences did not reach strong significance. Other correlations between PA indices and predictive SES-variables were not significant [15]
Trinh et al., 2008	Identify PA patterns and factors associated with “insufficient” levels of PA for health in adults. *Questionnaire*	*n* = 1906 M: 884 F: 1022 Age: 25–64 VIETNAM	Education: + Income: + Occupation: + Neighborhood: + Other: household	Income and household wealth index significantly related to insufficient PA. Monthly income associated with insufficient PA. However, the household wealth index shows a significant association from the middle quintiles onwards, with people from wealthier households having greater risks of insufficient PA; especially among M. Tests for trend across income and household wealth index also confirmed this observation. The results across both genders show this association, but no significant association in W [48]
Naseer et al., 2013	Identify sex-based differences in the perception of benefits and barriers toward exercise and determine the sex- and age-based differences in the level of PA in adult residents of Karachi. *Questionnaire*	*n*= 300 M: 125 F: 175 Age: 18< PAKISTAN	Education: + Income: + Occupation: − Neighborhood: − Other: work status	PA was highest in M w/income less than 6000 Pakistan rupees. PA is lowest in M w/income between 6000–16,000 Pakistan rupees. F: less fluctuation in results. Education not reported [49]
Vaidya & Krettek, 2014	Measure PA in LTPA, OPA + TPA in a peri-urban community and assess its variations across different sociodemographic correlates. *Questionnaire*	*n* = 640 M: 175 F: 465 Age: 25–59 NEPAL	Education: + Income: − Occupation: + Neighborhood: − Other: −	Low PA was lowest among males who had studied up to grade 4 (23.3%). Compared with informal education, PIA was ×3 higher in individuals educated up to high school or more. Those who worked in agro-based jobs had the highest Total PA. In terms of Total PA, inadequate PA was more likely in government employees, self-employed individuals, and housewives [50]

^1^ The symbols +/− indicates whether the particular SES measure is used in the study (+) or not (−).

**Table 4:** Studies investigating PA in adults across SES. No relationship.

Study	Aim and *Study Design*	Sample	Measures of SES ^1^	Outcome/Conclusion
Schneider et al., 2009	Group clusters that exhibit specific health behavior patterns regarding (…) and PA. *Phone interview (questionnaire)*	*n* = 2002 M: 982 F: 1020 Age: 50–70 GERMANY	Education: + Income: + Occupation: + Neighborhood: − Other: −	No significant characteristic of the inactive cluster related to SES [51]
Molina-García et al., 2010	Examine psychosocial and environmental correlations of TPA to university and explore its associations with overall PA among students. *Survey*	*n* = 518 M: unknown F: unknown Age: 22.4 SPAIN	Education: − Income: − Occupation: − Neighborhood: − Other: (low→high)	SES was not a significant correlate of active commuting to university [17]
Chen et al., 2011	Explore the determinants influencing adults’ LTPA in a city in southern Taiwan. *Questionnaire*	*n* = 762 M: 359 F: 403 Age: 40–67 TAIWAN	Education: + Income: − Occupation: + Neighborhood: − Other: marital status	Indicators of high SES were positively associated with participation in exercise/sports, but no significant correlation was found [52]

^1^ The symbols +/− indicates whether the particular SES measure is used in the study (+) or not (−).

**Table 5:** Studies investigating PA in adults across SES. Mixed relationships.

Study	Aim and *Study Design*	Sample	Measures of SES ^1^	Outcome/Conclusion
Borrell et al., 2000	Describe social class inequalities in health related behaviors. *Interview survey*	*n* = 4171 M: 1942 F: 2229 Age: 14–65+ SPAIN	Education: − Income: − Occupation: + Neighborhood: − Other: −	Less than 5% of M and W in class 1 (highest SES) declared that they usually performed intense PA in contrast with 11.5% of M and 8.6% of W in class 5, an association that persisted in the multivariate analysis. People of classes 1&2 were more likely to engage in usual PA classified as “light or none” than lower classes. For LTPA the situation was reversed, particularly in M, as a greater proportion of the lower classes did not engage in PA three or more times per week. In the multivariate analysis, the association was not significant. In W, there was no clear trend. Engaging in usual PA as “light or none” in M decreased with lowering class [53]
Livingstone et al., 2001	Evaluate habitual levels of PA. *Questionnaire*	*n* = 1369 M: 655 F: 714 Age: 18–64 IRELAND	Education: − Income: − Occupation: + Neighborhood: − Other: −	Professional/skilled non-manual M engage in less total and OPA than M from other social groups. Reverse in W. HPA by M were broadly similar across social class groupings but W in skilled manual/partly skilled/unskilled occupations spent more time in these HPA than W from other social groups. Differences in time spent in vigorous active recreation by M were reported, but none was significant. Approximately 2× difference in the range of time spent in vigorous active recreation by the W (0.7 ± 0.9 h·week^−1^ skilled manual vs. 1.2 ± 2.0 h·week^−1^ skilled non-manual). W in professional/skilled non-manual groups spent significantly more time in these pursuits than W in other social class groupings [54]
Popham & Mitchell, 2007	Investigate further associations between SES position and overall PA levels and specific types of PA. To investigate the role of employment status and health. *Questionnaire*	*n* = 5287 M: 2346 F: 2941 Age: 25–64 SCOTLAND	Education: + Income: − Occupation: + (parent’s and own) Neighborhood: − Other: housing tenure	Increasing accumulated socioeconomic disadvantage was associated with higher rates of low or no PA. For M, this association largely disappeared after adjustment for employment status and health, while among W the differences were reduced. Although low SES was associated with higher rates of OPA, the most disadvantaged did not have the highest rate. However, after adjustment for employment status (especially) and health, a clearer social gradient in OPA emerged in which relative rates of OPA increased with increasing disadvantage. Low SES was associated with low rates of participation in brisk walking, sport and exercise and heavy manual leisure. SES differences in these PA were not greatly changed after adjustment for health and employment status [55]
Allender et al., 2008	Examine relative contribution of OPA to English adults’ meeting PA recommendations. *Cross-sectional survey, individual interviews*	*n* = 13,974 M: 6237 F: 7737 Age: 16–75+ ENGLAND	Education: + Income: + Occupation: + Neighborhood: − Other: −	Education: OPA included, M w/any qualification were more likely to meet the PA guideline than those w/a degree or higher or the no qualification group. OPA removed; those w/any qualification or a degree qualification or higher were more likely to meet the guideline than the no qualifications group. Occupation: OPA included; unskilled manual, semiskilled manual and skilled manual W were more likely to meet the PA guideline than the professional group. Not significant when OPA was removed from the analysis [56]
Jurakic et al., 2009	Determine the PA level in different domains of everyday life. *Questionnaire*	*n* = 1032 M: 500 F: 532 Age: 15+ CROATIA	Education: + Income: + Occupation: − Neighborhood: − Other: settlement	Total PA was inversely related to the size of settlements. OPA domain was also inversely related to the size of settlements. Furthermore, TPA was inversely related to household income, while PA in HPA was positively related to age and inversely related to the size of settlements and educational level. Finally, LTPA was positively related to the size of settlements and to household income [57]
Molina-García et al., 2014	Describe differences in energy exposure in active commuting to university by transport mode in students and examine sociodemographic associations with energy exposure. *Questionnaire*	*n* = 518 M: 209 F: 309 Age: mean 22.4 SPAIN	Education: − Income: − Occupation: − Neighborhood: − Other: subjective definition	Low SES-students walked more but biking was significantly higher in the high SES group than the medium SES group [18]
Golubic et al., 2014	Describe PA and sedentary behavior and examine the variation of PA-sub-components by key health-related, anthropometric, and socio-demographic factors as well as prior PA. *Questionnaire, heart rate and move sensing*	*n* = 1787 M: 862 F: 925 Age: 60–64 GREAT BRITAIN	Education: + Income: − Occupation: + Neighborhood: − Other: employment status	For those still working, M in manual work had higher PA energy expenditure (14%), than non-manual workers, but values for W did not differ. In W, PA energy expenditure were greater with higher education. PA energy expenditure from questionnaire was higher in full-time employed than in those who were employed part time or retired. PA energy expenditure were greater in those in manual than non-manual occupations in M, but not significant in W. W with higher education had higher PA energy expenditure than those with lower, but the opposite patterns were observed in M [21]
Hawkins et al., 2004	Describe the prevalence of self-reported moderate/vigorous PA. *Questionnaire*	*n* = 40,261 M: 18,375 F: 21,406 Age: 20–55+ USA	Education: + Income: − Occupation: − Neighborhood: − Other: −	Subjects with education beyond high school were less likely to meet the moderate PA guideline than those with less education. The younger, M, and better educated were most likely to achieve the vigorous PA guideline before and after adjustment for potential confounding variables [58]
Berrigan et al., 2006	Explore inclusion of *non-leisure-time walking and bicycling* (NLTWB) used for transportation on the prevalence of adherence to PA recommendations and the magnitude of apparent disparities in adherence for Californian adults. *Phone survey*	*n* = 55,151 M: 22,930 F: 32,221 Age: 18–≥70 USA	Education: + Income: + Occupation: − Neighborhood: − Other: −	Adherence based on LTPA increased with education and income level. By contrast, adherence based on NLTWB decreased with education and income. Logistic regression confirmed the presence of significant effects of(…) education, and income on adherence based on LTPA and the prevalence of adherence based on LTPA and NLTWB combined. In multivariate models, (…) education, and income, were associated with adherence based on NLTWB alone. LTPA increases as education and income levels increase but NLTWB decreases [16]
Lee & Levy, 2011	Examine PA in multiple contexts and blood pressure across gender and income among older adults living independently. *Questionnaire*	*n*= 372 M: 128 F: 244 Age: 60+ USA	Education: + Income: + Occupation: − Neighborhood: − Other: −	M at low income levels reported greater HPA than M at high income levels. For W, no differences by income level in HPA were seen. Income level alone also made a significant contribution to differences seen in HPA however these effects appear to have been overridden by the significant interaction. Income level significantly contributed to differences seen in total LTPA with those at low income levels reporting less LTPA than those with higher income levels [59]
Florindo et al., 2009	Estimate the prevalence of and identify factors associated with LTPA, TPA, OPA and HPA. *Questionnaire*	*n* = 1318 M: 652 F: 666 Age: 18–65 BRAZIL	Education: + Income: − Occupation: − Neighborhood: − Other: −	Higher education level was negatively associated with low level of LTPA, while it was positively associated with low activity in both OPA and HPA [60]
Bicalho et al., 2010	Estimate the PA level and its association with SES factors in adults living in rural areas. *Questionnaire*	*n* = 567 M: 275 F: 292 Age:18–60 BRAZIL	Education: + Income: − Occupation: − Neighborhood: − Other: marital status	There was an inverse relationship between education and the percentage of participants performing 150 min. at work. Education had an inverted U-shaped association with the practice of HPA, in the total population and in W. M (*p* = 0.133). LTPA was more frequent in individuals with greater education for total, M, and W. M with higher education were the least active in the TPA domain. (W and total: not significant) [61]
Del Duca et al., 2013	Estimate the prevalence and sociodemographic indicators associated with PIA LTPA, TPA, OPA and HPA in adults. *Questionnaire*	*n* = 1720 M: 769 F: 951 Age: 20–59 BRAZIL	Education: + Income: + Occupation: − Neighborhood: − Other: −	LTPA: those with lower education and lower income had higher probability of PIA. TPA: higher income presented higher prevalence of PIA. OPA: higher education and income, more PIA. HPA: Higher education and higher income; higher prevalence of PIA [62]
Linetzky et al., 2013	Evaluate how SES gradients in non-communicable diseases and non-communicable disease-related risk factors change over time (2005–2009). *Questionnaire*	*n* = 41,392/34,732 M:17,827/15,028 F:23,565/19,704 Age: 43.3/43.6 ARGENTINE	Education: + Income: + Occupation: − Neighborhood: − Other: −	In 2005, M with low education (OR = 0.65, 95% CI = 0.50–0.85) and medium education (OR = 0.79, 95% CI = 0.67–0.93) were less likely than males with high education to be physically inactive. In 2009, the direction of the gradient switched direction. By 2009, W with low education (OR = 1.57, 95% CI = 1.34–1.84) and medium education (OR = 1.18, 95% CI = 1.06–1.32) were more likely than women with high education to be physically inactive [63]
Giles-Corti et al., 2002	Examine spatial access to recreational facilities and perceptions of the neighborhood environment and PA levels by the SES of area of residence. *Survey*	*n* = 1803 M: 580 F: 1223 Age: 18–59 AUSTRALIA	Education: + Income: + Occupation: − Neighborhood: + Other: work outside home, access to motor vehicle	No difference between the two SES areas in walking overall, but the types of walking differed significantly. Compared with high SES areas, walking for transport was 33% more prevalent in walkers from low SES areas and walking for recreation was 21% lower. Participation in vigorous PA was 24% lower for those living in low SES areas compared with those in high SES areas and participation in light to moderate PA was 16% lower. On average, compared with those living in high SES areas, those living in low SES areas who walked for transport did so for nearly 1 more hour per fortnight more. Although the difference in walking occasions did not reach significance, in low SES areas transport walkers walked on nearly two more occasions per fortnight. Respondents living in low SES areas were 26% less likely to do sufficient PA compared w/those living in high SES areas. The odds of reaching high levels of vigorous PA were also near 50% lower [64]
Proper et al., 2006	Examine the influence of neighborhood and individual SES on OPA. *Questionnaire*	*n* = 1236 M: 470 F: 766 Age: 20–65 AUSTRALIA	Education: + Income: + Occupation: − Neighborhood: + Other: −	Neighborhood SES and individual SES were independently inversely related to absolute and relative amount of OPA. Significant interactions between neighborhood SES and level of educational attainment in the contribution of total and vigorous OPA to total PA were found [65]
Kahan, et al., 2005	Evaluate levels of LTPA, OPA, sports PA and correlate them with SES and health factors *Questionnaire*	*n* = 406 M: 173 F: 211 Age: 20–65 ISRAEL	Education: + Income: + Occupation: − Neighborhood: − Other: −	OPA level decreased with level of education, whereas sports PA increased. The sports index was also directly correlated with monthly income status: income 5000< NIS (4 NIS equaled U.S. $1.00) was associated with a significantly higher sports PA index and lower OPA index. Regression models showed that the lower the level of education, the greater the degree of OPA and the lower the degree of sports PA. The higher the income, the greater tendency to less OPA but more at sports PA [66]
Khaing Nang et al., 2010	Evaluate the characteristics of individuals participating in different PA domains. *Questionnaire*	*n* = 4750 M: 2280 F: 2470 Age: 18–60 SINGAPORE	Education: + Income: + Occupation: − Neighborhood: − Other: work, house	A higher SES was associated with a higher likelihood of participating in LTPA. OPA was higher in those with low SES. TPA was lower for those with higher SES. HPA was lowest for those with higher SES. Participants with a higher SES had more LTPA, but less OPA, TPA and HPA resulting in lower overall PA [67]
Saito et al., 2013	Examine the association of 3 types of PA and their associations with individual and neighborhood environmental factors among middle-aged and elderly Japanese. *Questionnaire*	*n* = 1940 M: 943 F: 997 Age: 40–69 JAPAN	Education: + Income: + Occupation: − Neighborhood: + Other: employment, number of children	Not working increased and number of children in the household decreased the odds of all three types of PA (not all significant). Economic status increased the odds of moderate-to-vigorous LTPA but decreased the odds of transport-related walking. High education increased the odds of moderate-to-vigorous LTPA. Owing motor vehicles increased the odds of engaging in moderate-to-vigorous LTPA other than walking [68]
Talaei et al., 2013	Investigate PA by SES and sex in an Iranian adult population. *Questionnaire*	*n* = 6622 M: 3221 F: 3401 Age: mean 45.2 IRAN	Education: + Income: + Occupation: + Neighborhood: − Other: employment	LTPA: higher for high SES participants than middle and low SES for both M and W. OPA: W: no significant difference between low and high SES. M: less in high than middle and low SES. HPA: W: significantly different (*p* < 0.001) higher in middle than high and low SES: it was lower in high than low SES. M: No significant differences found. TPA: M and W: No significant differences [69]
Ying Chan et al., 2014	Examine the association between socio-demographic factors and PIA by gender. *Questionnaire*	*n* = 33,949 M: 15,205 F: 18,744 Age: 18–65+ MALAYSIA	Education: + Income: + Occupation: − Neighborhood: − Other: employment	PIA in M increased with increasing income, but not in W. The widow/widower/divorcee, non-working group, and those with no formal education were found to have high PIA in both M and W [70]

PA: physical activity; LTPA: leisure time physical activity; OPA: occupational physical activity; HPA: household physical activity; TPA: transporting physical activity; PIA: physical inactivity; SES: socioeconomic status; SEP: Socioeconomic position; M: men; W: women. ^1^ The symbols +/− indicates whether the particular SES measure is used in the study (+) or not (−).
